# A rare case of obstructive jaundice: endoscopic diagnosis and treatment

**DOI:** 10.1055/a-2675-4928

**Published:** 2025-09-09

**Authors:** Xianhong Zhao, Xujie Xi, Jialiang Yin, Yingxiu Zhang, Meishun Lin, Haiyan Zhang, Wenbin Wu

**Affiliations:** 1 74715 Department of Gastroenterology, The Second Affiliated Hospital of Guangzhou University of Chinese Medicine, Guangzhou, China

Obstructive jaundice is a common health problem. It can be either intrahepatic or extrahepatic according to the location of onset. Extrahepatic obstructive jaundice can be caused by obstruction of the common bile duct by stones, strictures, inflammatory edema, tumors, and roundworms. Biliary atresia, bile duct injury, and pancreatic lesions are other causes often met with in clinical practice 1 . Some rare causes of obstructive jaundice have also been reported, such as the aortoduodenal syndrome and Mirizzi syndrome 2 3 . Herein, we report a rare case of obstructive jaundice from a cause that has never been previously reported.

A 74-year-old man with chief complaints of fever, abdominal pain, and vomiting came to our hospital for treatment. He had undergone Billroth II surgery for gastric tumor 10 years previously. Computed tomography previously performed in another hospital showed inflammatory stenosis of the lower common bile duct accompanied by inflammation and exudation around the pancreas and afferent loop. Relevant blood test results, including for high-sensitivity C-reactive protein (hsCRP), white blood cells, total bilirubin and direct bilirubin, liver enzymes, and amylase, were abnormally elevated, indicating the presence of obstructive jaundice and biliary pancreatitis. Therefore, we planned to perform emergency endoscopic retrograde cholangiopancreatography (ERCP) for biliary drainage. We used a transparent cap on the front end of the gastroscope to enter the afferent loop and searched for the duodenal papilla. During the process of advancing, a huge round yellow foreign body was found in the afferent loop. The small intestinal mucosa around the foreign body had developed ulcers and necrosis due to compression. A foreign body forceps, polyp snare, and stone retrieval basket were used to attempt extraction of the foreign body. However, all attempts failed because the foreign body was hard and round and tightly wedged in the surrounding intestinal wall. In the end, we replaced the snare with a harder and sharper one, and removed the foreign body by cutting it into small pieces ( Fig. 1 ). However, what surprised us was that, while advancing the gastroscope towards the blind end of the afferent loop, we found another foreign body of the same type. In view of the difficulty and long duration of the endoscopic operation, selective endoscopic treatment was scheduled.

**Fig. 1:**
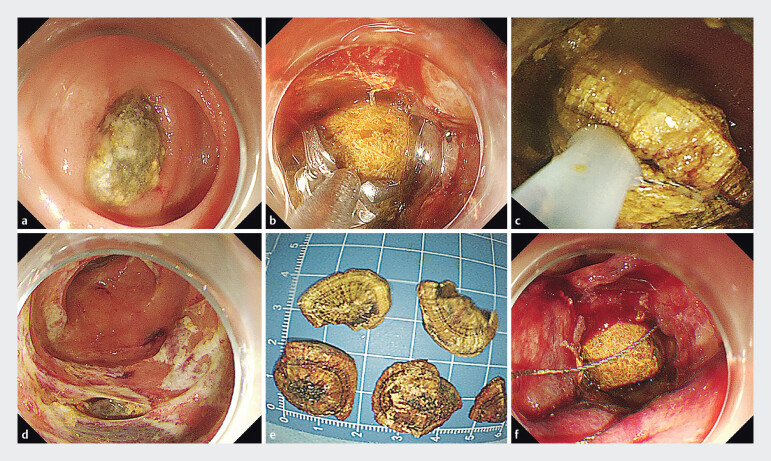
The first endoscopic procedure, undertaken to perform emergency biliary drainage in a 74-year-old man with altered gastric anatomy after Billroth II surgery. **a** A huge round yellow foreign body was found in the afferent loop. **b** A foreign forceps and polyp snare were used to attempt extraction of the foreign body, but this failed. **c** A harder and sharper snare was used to cut the foreign body into small pieces. **d** The small intestinal mucosa had developed ulcers and necrosis due to compression. **e** The extracted fragments of the foreign body. **f** Another, similar foreign body was found on the anal side.

The next day, endoscopic treatment was performed with tracheal intubation and general anesthesia. This was a more difficult procedure because the intestinal mucosa became more swollen as the gastroscope advanced closer to the blind end of the afferent loop. Attempts at extraction using a snare, stone retrieval basket, and guidewire-assisted stone extraction balloon failed. Guidewire-assisted piecemeal resection as previously reported was also tried 4 , but to no avail. In the end, we used a mucosal incision knife to perform electrical cutting on the foreign body ( Fig. 2 ). The small fragments of the foreign body were extracted using a the retrieval basket ( Video 1 ). A total of six similar foreign bodies were extracted using this method. The last foreign body was located in the blind end of the afferent loop. The mucosa around the foreign body was significantly swollen and the structure of the duodenal papilla was indistinct, which was considered to be the cause of the obstructive jaundice ( Fig. 3 ). No endoscopy-related adverse event occurred. The patient’s blood indicators gradually returned to normal and he was discharged from hospital after 1 week ( Fig. 4 ). In consultation with members of the patient’s family, the foreign bodies were identified as durian kernels.

**Fig. 2:**
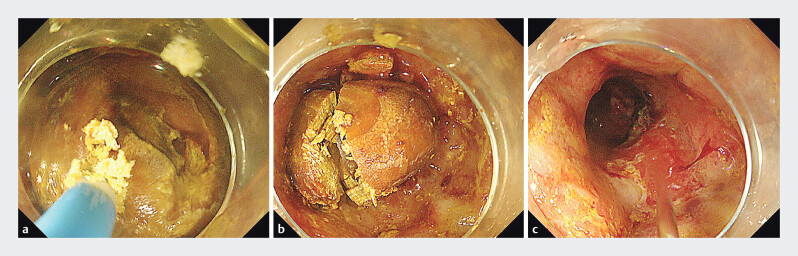
The second endoscopic procedure, removing a second and subsequent foreign bodies. **a** A mucosal incision knife was used to perform electrical cutting on the foreign body. **b** The foreign body was cut into fragments. **c** The mucosa was swollen and the structure of the duodenal papilla was indistinct.

**Fig. 3:**
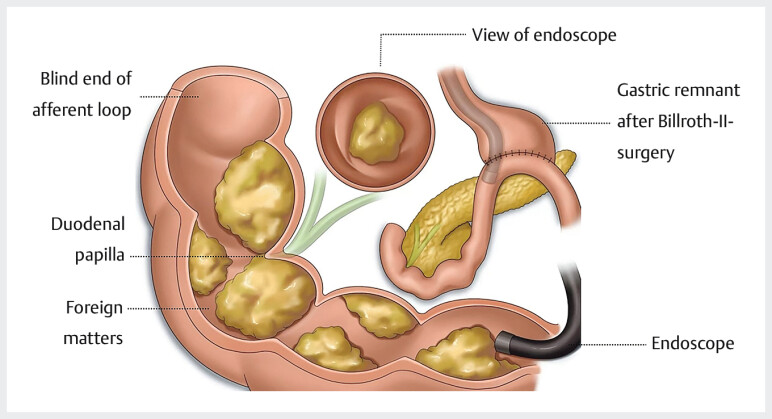
Diagrammatic illustration of the anatomical location of the foreign bodies and the duodenal papilla.

**Fig. 4:**
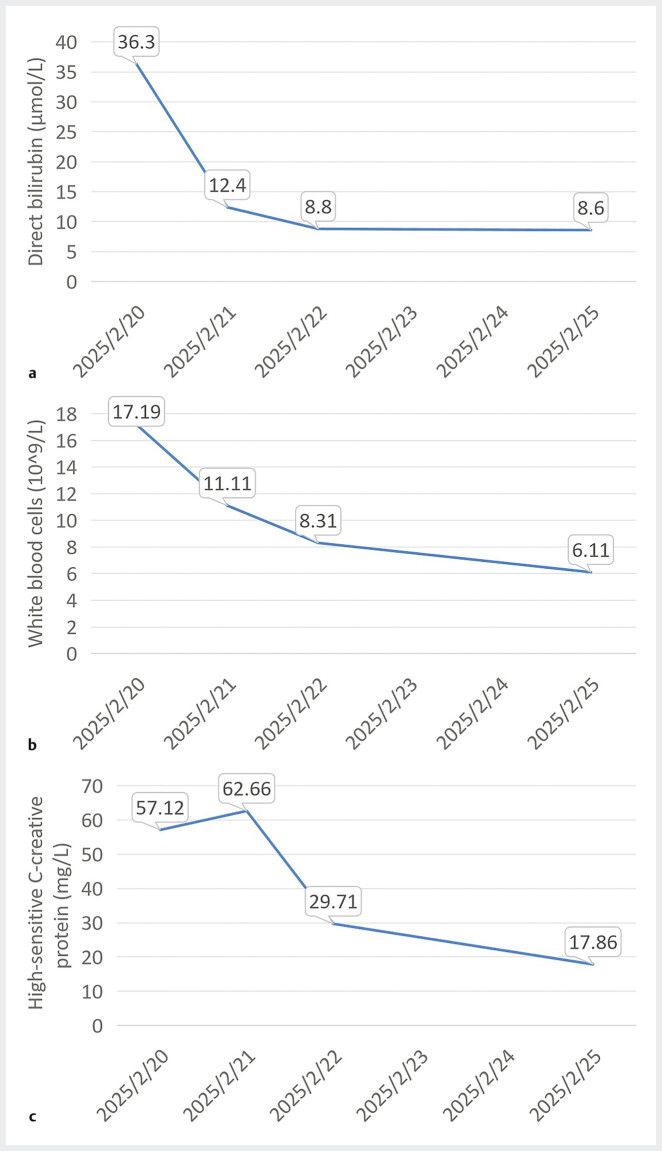
The patient’s blood indicators gradually returned to normal in the immediate postoperative period.

Attempting by various means to extract foreign bodies in the afferent loop of a patient with altered gastric anatomy after Billroth II surgery.Video 1

This is the first case report of obstructive jaundice caused by multiple foreign bodies in the afferent loop in a patient with altered gastric anatomy after Billroth II surgery. Electrical cutting into small fragments using a mucosal incision knife may be an alternative method for such huge foreign bodies.

Endoscopy_UCTN_Code_CCL_1AZ_2AN

## Notes

Endoscopy E-Videos https://eref.thieme.de/e-videos
*E-Videos* is an open access online section of the journal *Endoscopy* , reporting on interesting cases and new techniques in gastroenterological endoscopy. All papers include a high-quality video and are published with a Creative Commons CC-BY license. Endoscopy E-Videos qualify for HINARI discounts and waivers and eligibility is automatically checked during the submission process. We grant 100% waivers to articles whose corresponding authors are based in Group A countries and 50% waivers to those who are based in Group B countries as classified by Research4Life (see: https://www.research4life.org/access/eligibility/ ). This section has its own submission website at https://mc.manuscriptcentral.com/e-videos .

## References

[1] Liu JJ Sun YM Xu Y Pathophysiological consequences and treatment strategy of obstructive jaundice World J Gastrointest Surg 2023 15 1262 1276 10.4240/wjgs.v15.i7.1262 37555128 PMC10405123

[2] Ur Rahman A Joseph C Green J Unique case of abdominal aortic aneurysm causing obstructive jaundice and duodenal obstruction J R Coll Physicians Edinb 2023 53 30 34 36708217 10.1177/14782715231152729

[3] Liu P Tan XZ Wan R An unusual cause of obstructive jaundice Gastroenterology 2023 164 196 197 10.7759/cureus.14307 35932887

[4] Ao J Huang S Liao S Guidewire-assisted piecemeal resection of a giant gastric tumor Endoscopy 2024 56 E13 E14 10.1055/a-2218-3297 38194981 PMC10776273

